# Renal insufficiency was correlated with 2-year mortality for rural female patients with ST-segment elevation acute myocardial infarction after reperfusion therapy: a multicenter, prospective study

**DOI:** 10.1186/s12872-015-0174-2

**Published:** 2015-12-24

**Authors:** Yuan Gao, Daming Jiang, Bo Zhang, Yujiao Sun, Lina Ren, Dandan Fan, Guoxian Qi

**Affiliations:** Department of Cardiology, First Affiliated Hospital of China Medical University, Shenyang, Liaoning 110001 China; Department of Cardiology, Dandong Center Hospital, Dandong, Liaoning 118000 China; Department of Cardiology, First Affiliated Hospital, Dalian Medical University, Dalian, Liaoning 116011 China; Department of Geriatric Cardiology, First Affiliated Hospital of China Medical University, Shenyang, Liaoning 110001 China

**Keywords:** ST-segment elevation, Myocardial infarction, Renal insufficiency, Risk factors

## Abstract

**Background:**

Renal insufficiency (RI) following ST-segment elevation acute myocardial infarction (STEMI) is associated with a worse clinical prognosis. We investigated the impact of RI on long-term mortality in rural female patients with STEMI and evaluated prognostic factors.

**Methods:**

A prospective cohort study of 436 consecutive rural female patients who were successfully treated with reperfusion therapy for STEMI between May 2009 and August 2011 in secondary care hospitals in Liaoning province northeastern China and followed up for 2 years. Patients were divided into three groups by estimated glomerular filtration rate (eGFR): Normal group, eGFR ≥90 mL/min/1.73 m^2^ (*n* = 233). Moderate group, eGFR 60–90 mL/min/1.73 m^2^ (*n* = 108). RI group, eGFR <60 mL/min/1.73 m^2^ (*n* = 95). The primary outcome was 2-year mortality.

**Results:**

During follow-up (mean 741 ± 118 days), the RI group had a significantly higher mortality than the other groups (24.21 % vs. 6.87 % and 10.19 %, *p* < 0.001). The RI group had significantly higher hospital mortality (7.37 % *p* = 0.045 vs. Normal group). RI increased the risk of hospital mortality (hazard ratio (HR) 1.832, 95 % CI 1.017–3.091, *p* = 0.033), and increased the risk of 2-year mortality (HR 3.872, 95 % CI 2.004–6.131, *p* < 0.001). Multivariate analysis showed eGFR <90 ml/min/1.73 m^2^ and age ≥75 years as independent predictors of mortality at 2 years. In detail these were eGFR 60–90 ml/min/1.73 m^2^ with HR 2.081, 95%CI 1.250–2.842, *p* < 0.001; eGFR <60 ml/min/1.73 m^2^ with HR 3.872, 95%CI 2.004–6.131, *p* < 0.001; age ≥75 with HR 1.461, 95%CI 1.011–1.952, *p* = 0.024.

**Conclusions:**

RI had a powerful correlation with long-term mortality for rural female patients with STEMI after reperfusion therapy.

## Background

At present the incidence of chronic kidney disease is rapidly increasing [1]. Nearly 30 % of patients with ST-segment elevation acute myocardial infarction (STEMI) have combined renal insufficiency (RI) [2]. Widely used early reperfusion therapy, including emergency primary percutaneous coronary intervention (PCI) or thrombolysis therapy has beneficial effects for STEMI [3, 4]. However, RI following STEMI is associated with a worse clinical prognosis [5–7], a 6 to 11-fold increase in hospital risk of death [8], and a 1.76- to 6.18-fold 7-month risk of death [6]. Unfortunately, most STEMI patients with RI are excluded from randomized trials. Renal insufficiency may lead to alteration in lipid metabolism, vascular endothelial injury and dysfunction, trigger the inflammatory response, coagulation and oxidative stress and increase atherosclerosis, by the sympathetic, neurohormonal pathway and renin angiotensin aldosterone axis activation [9–11].

Most clinical studies into myocardial infarction involve only a minority of female patients. For example women accounted for 29.6 % of the total enrolled patients in the Korea acute myocardial infarction registry study [7]. This is of concern because acute myocardial infarction mortality is higher in females than males and while there have been declines in the risk of death in men; the rate in women remains fairly constant [12]. The risk of in hospital mortality after primary PCI is also significantly higher for females than males [13], and female patients with STEMI show significantly greater death rates than males [14], with younger females at much higher risk than males of the same age [15].

In China the rates of mortality due to cardiac disease are growing, and while they are highest in urban areas, the rate in rural areas is increasing more rapidly [16]. Therefore, female patients with STEMI complicated by RI who reside in rural areas are an often neglected population that may be at high risk of death resulting from their condition. Little is known of the impact of RI on the prognosis in rural female patients with STEMI regardless of reperfusion therapy in Liaoning province in northeastern China.

The objective of this study was to determine the association between RI and the risk of death in STEMI patients successfully treated with PCI or thrombolytic therapy. We hypothesized that RI would be associated with higher 2-year mortality. The results of our prospective cohort study provide convincing evidence of this association in a real world situation.

## Methods

### Subjects

This was a prospective, multicenter study conducted at 16 hospitals in the Liaoning Province of northeast China from May 2009 to August 2011. The 16 hospitals were: First Affiliated Hospital, China Medical University; First Affiliated Hospital, Dalian Medical University; Changtu xian People’s Hospital; Fuxin Mongolian Autonomous County People’s Hospital; Yixian People’s Hospital; Benxi Steel Company Hospital; Fushun Coal Mining Administration Hospital; Chaoyang Center Hospital; Fuxin Center Hospital; Zhuanghe Center Hospital; Wafangdian Center Hospital; Pulandian Center Hospital; Donggang Center Hospital; Dashiqiao Center Hospital; Zhangwu xian People’s Hospital; Kuandian xian Center Hospital.

This study was conducted in accordance with the declaration of Helsinki, and was conducted with approval from the Ethics Committee of China Medical University. Written informed consent was obtained from all participants.

We enrolled 479 consecutive rural female STEMI patients from May 2009 to August 2011 from all of the centers. The inclusion criteria were: (1) STEMI was diagnosed according to European Society of Cardiology (ESC) criteria [3]; (2) it was the first time STEMI was diagnosed; (3) all patients were given primary PCI treatment within 12 h or thrombolytic therapy within 6 h after symptom onset. The exclusion criteria were: (1) acute myocardial infarction patients with acute kidney injury (AKI); (2) patients undergoing dialysis treatment. (3) patients whose non-infarct-related artery was treated during primary PCI; (4) PCI was undertaken after thrombolytic therapy.

Acute renal failure was diagnosed by an increase in serum creatinine levels of 50 % or an absolute increase of ≥26.5 μmol/l in 48 h [17].

Demographic and basic clinical data were obtained from all patients, which included age, gender, body mass index and cardiovascular risk factors. Additional clinical data including clinical laboratory tests, coronary imaging data, therapeutic strategies and adverse cardiac events were collected by trained personnel.

### eGFR measurement

Kidney function was measured by estimated glomerular filtration rate (eGFR), calculated using the Chronic Kidney Disease Epidemiology Collaboration (CKD-EPI) Modification of Diet in Renal Disease (MDRD) equation [18] [CKD-EPI formula If female and if serum creatinine (Scr) ≤0.7 mg/dL:

$$ \mathrm{C}\mathrm{K}\mathrm{D}\hbox{-} \mathrm{E}\mathrm{P}\mathrm{I}=144\times \mathrm{S}\mathrm{c}\mathrm{r}\left(\mathrm{mg}/\mathrm{dL}\right)/0.{7}^{\hbox{-} 0.329}\times 0.99{3}^{\mathrm{age}\left(\mathrm{years}\right)} $$

If female and if Scr > 0.7 mg/dL:

$$ \mathrm{C}\mathrm{K}\mathrm{D}\hbox{-} \mathrm{E}\mathrm{P}\mathrm{I}=144\times \mathrm{S}\mathrm{c}\mathrm{r}\left(\mathrm{mg}/\mathrm{dL}\right)/0.{7}^{\hbox{-} 1.209}\times 0.99{3}^{\mathrm{age}\left(\mathrm{years}\right)}. $$

Renal insufficiency (RI) was defined as eGFR <60 mL/min/1.73m^2^according to the Kidney Disease: Improving Global Outcomes (KDIGO) guidelines [19] eGFR were calculated by the Scr examination results immediately after admission.

The patients were divided into 3 groups based on their eGFR values; the Normal group (high eGFR group, eGFR ≥ 90 mL/min/1.73 m^2^, *n* = 233), the Moderate group (middle eGFR group, 60 mL/min · m^2^ < eGFR <89 mL/min/1.73 m^2^, *n* = 108) who had moderate RI, and the RI group (low eGFR group, eGFR <60 mL/min/1.73 m^2^, *n* = 95).

### Successful PCI

PCI was considered to be successful if the infarct-related artery residual stenosis was <10 %, and the Thrombolysis in Myocardial Infarction (TIMI) flow level reached 3 [20]. All patients were estimated immediately after PCI.

### Successful thrombolytic therapy

1.5 million Units of urokinase were administrated to patients by intravenous infusion within 30 min for thrombolytic therapy.

Thrombolytic therapy was considered successful if more than 2 of 4 criteria were met [9]: ST segment resolved by ≥ 50 % by electrocardiogram (ECG) within 2 h, chest pain disappeared within 2 h; reperfusion arrhythmia occurred; serum myocardial enzyme peaks were detected in advance.

### Medication

All patients received the recommended standard management for STEMI, including 300 mg loading dose of aspirin and clopidogrel after admission, aspirin, clopidogrel, low-molecular-weight heparin, beta-blockers, statins and angiotensin-converting enzyme (ACE) inhibitors/ angiotensin receptor antagonist (ARB) as appropriate. The details of the medications are included in Appendix 1.

### Definition of risk factors

Abnormal body mass index (BMI) was defined as BMI ≥ 25 kg/m [2, 21]. Diabetes was diagnosed by previous medical history or fasting glucose ≥ 7.0 mmol/L and/or 2 h plasma glucose level ≥11.1 mmol/l (measured after 75 g oral glucose load). Hypertension was diagnosed by previous medical history or systolic blood pressure ≥ 140 mmHg and/or diastolic blood pressure ≥ 90 mmHg after admission. Hypercholesterolemia was diagnosed by previous medical history or low-density lipoprotein (LDL) cholesterol ≥ 2.6 mmol/L and/or non high-density lipoprotein (HDL) cholesterol ≥3.3 mmol/L. Definition of contrast-induced nephropathy (CIN) was an increase in serum creatinine ≥ 0.5 mg/dL, occurring 48 h after exposure to contrast media [22]. Current smoker was defined as smoking >300 cigarettes/year.

### Outcomes

Major adverse cardiac events (MACE) included: death, recurrent myocardial infarction, target vessel revascularization and stroke.

Key bleeding end points were analyzed on the basis of global use of strategies to open occluded coronary arteries (GUSTO) criteria [23].

CIN, contrast-induced nephropathy was defined as either a greater than 25 % increase of serum creatinine or an absolute increase in serum creatinine of 0.5 mg/dL [24].

### Clinical examination and laboratory analysis

Patients underwent physical examination, ECG examination and fasting blood biochemical examination after admission including Scr, serum creatine kinase MB (CKMB), and cardiac troponin I (cTNI). Patients were examined for myocardial necrosis markers and by ECG once every 8 h in 72 h, then every 24 h they were examined again. Blood leukocyte counts were examined 24 h after admission and echocardiography 48 h after admission.

To ensure the standard of data collection, laboratory procedures, data management and coordination between the multiples centers involved in the study was up to our quality control all the clinicians involved in this study received uniform training before the research began.

### Follow up

The patients were followed up by telephone for 2 years; once per year; by the same doctor.

### Statistical method

Categorical data were expressed with absolute numbers and percentages and analyzed using the *χ*^2^ test. Continuous data with normal distribution were described with mean and standard deviation (SD) and median and interquartile range (IQR; 25th to 75th). Data among groups were compared using ANOVA. Further Student–Newman–Keuls (SNK) analyses were performed between the three groups. Non-normally distributed data among groups were compared using rank test.

Corresponding Kaplan-Meier curves with the log-rank test were constructed. Univariate analysis of eGFR, Scr, Microalbuminuria, age, gender, diabetes, hypertension, hyperlipidemia, Killip class, heart rate (HR), ejection fraction (EF), white blood cell (WBC) counts, and medication treatment were performed to determine the predictors for mortality. Multiple Cox proportional hazard model was used to estimate associations between significant factors identified in the univariate analysis. Hazard ratios (HR) and 95 % confidence intervals (CI) were calculated and p value <0.05 was considered statistically significant. All analyses were performed using SPSS version 19.0 (SPSS Inc., Chicago, IL, USA).

## Results

**Fig. 1:**
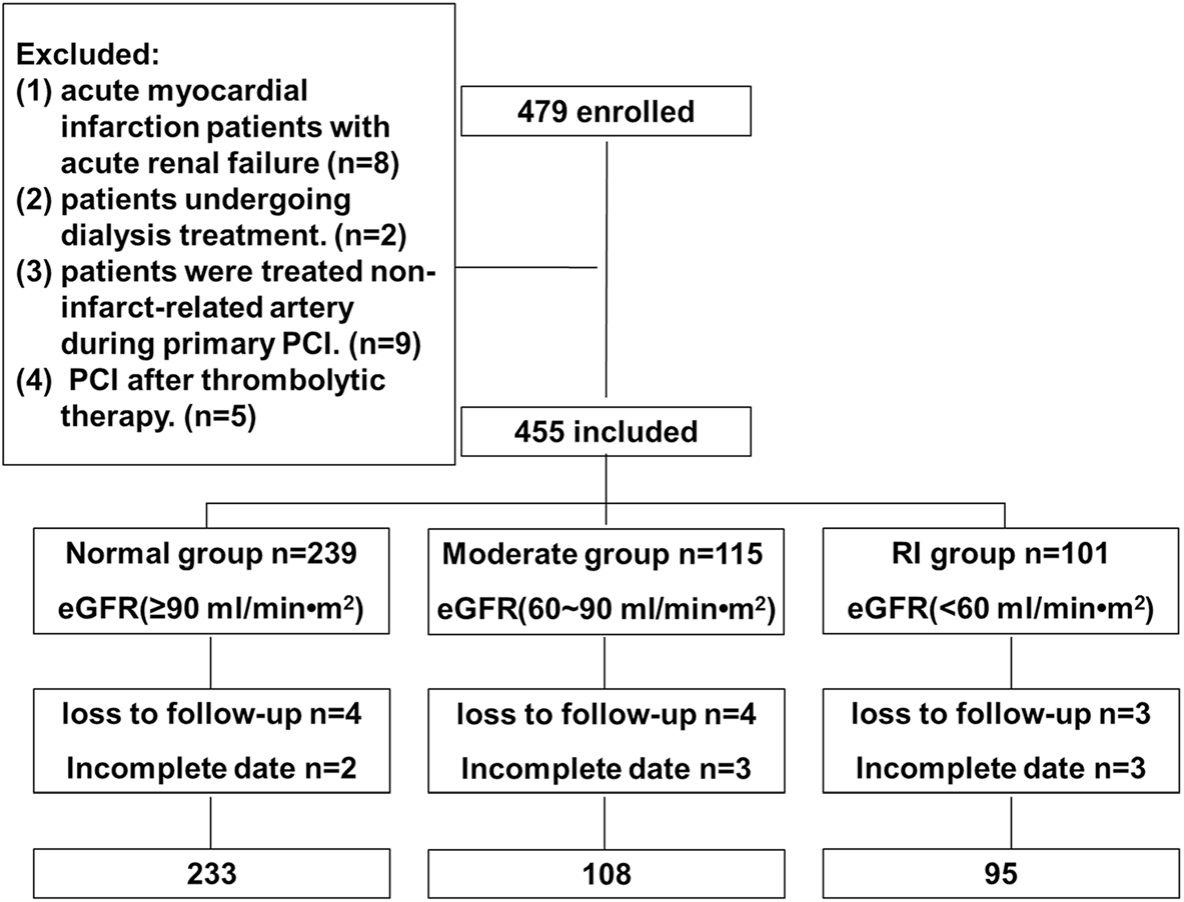
Flow chart of the selection of the study population and allocation into groups according to estimated glomerular filtration rate

The flowchart showing inclusion of patients in the study is presented in Fig. 1. In total 479 patients were enrolled in the study, 24 from these were excluded including 8 patients with incomplete data, 4 for whom clinical data were incomplete and 4 whose laboratory data were incomplete. Thus, 455 patients were included and 19 patients were lost to follow up. Finally 436 patients were included in the study 233 in the Normal group, 108 in the Moderate group, and 95 in the RI group (Fig. 1).

### Study sample and characteristics

The mean follow-up period was 741 ± 118 days. Among the 436 individuals in the final cohort, the mean age was 67.52 years, and 35.78 % had diabetes (Table 1). Elderly patients and those with hypertension and diabetes accounted for a high proportion of patients in the RI group. Median symptom to door time, door to balloon time and door to needle time were 183, 134 and 56 min, respectively. In the three groups the mean ages were 61.11 ± 8.42 years in the Normal group, 64.08 ± 6.91 years in the Moderate group and 75.57 ± 7.53 years in the RI group with a significant difference between all of the groups (*p* < 0.05). The diabetes rates were 29.61 % in the Normal group, 37.96 % in the Moderate group and 48.42 % in the RI group with a significant difference between the Normal and RI groups (*p* < 0.05). The hypertension rates were 32.19 % in the Normal group 44.44 % in the Moderate group and 52.63 % in the RI group with a significant difference between the Normal and RI group (*p* < 0.05). There were higher proportions in the RI group of Killip ≥ 2 at 21.05 % compared to 9.26 % (*p* < 0.05) in the Moderate group and 9.01 %, (*p* < 0.05) in the Normal group, longer hospital stay at 12.05 ± 5.74 days compared to 8.36 ± 5.11 days (*p* < 0.05) in the Moderate group and 8.53 ± 4.78 days (*p* < 0.05) in the Normal group, low EF values at 44.38 ± 13.05 % compared to 48.92 ± 14.02 % (*p* < 0.05) in the Moderate group and 50.11 ± 13.60 % (*p* < 0.05) in the Normal group, high Scr values at 116.67 ± 59.01 mmol/l compared to 85.47 ± 44.90 mmol/l (*p* < 0.05) in the Moderate group and 78.12 ± 31.13 mmol/l (*p* < 0.05) in the Normal group, and cTnI peak at 45.33 ± 15.26 ng/ml compared to 30.19 ± 18.73 ng/ml (*p* < 0.05) for the Moderate group and 31.06 ± 16.28 ng/ml for the Normal group (*p* < 0.05) (Table 1). There were 298 patients who underwent primary PCI and 138 thrombolytic therapy.

**Table 1:** Baseline characteristics

	Normal group (*n* = 233)	Moderate group (*n* = 108)	RI group (*n* = 95)	*χ* ^2^ /*F*	*p* value
Age (years)	61.11 ± 8.42	64.08 ± 6.91^a^	75.57 ± 7.53^ab^	114.59	<0.001
BMI (kg/m^2^)	24.51 ± 4.49	24.03 ± 3.98	23.95 ± 5.08	0.721	0.486
Killip ≥ 2	21 (9.01)	10 (9.26)	20 (21.05)^ab^	10.297	0.006
HR (bpm)	71.58 ± 23.40	73.29 ± 19.17	78.05 ± 25.18	2.823	0.061
Current smoker (%)	96 (41.20)	47 (43.52)	45 (47.37)	1.056	0.59
Diabetes (%)	69 (29.61)	41 (37.96)	46 (48.42)^a^	10.686	0.005
Hypertention (%)	75 (32.19)	48 (44.44)	50 (52.63)^a^	13.145	0.001
Hyperlipidemia (%)	79 (33.91)	36 (33.33)	29 (30.53)	0.799	0.671
Previous MI (%)	15 (6.44)	9 (8.33)	2 (2.11)	3.697	0.157
Previous PCI (%)	11 (4.72)	5 (4.63)	4 (4.21)	0.078	1
Previous stroke	2 (0. 86)	3 (2.78)	4 (4.21)	4.336	0.087
Peripheral vasculardisease (%)	3 (1.29)	1 (0.95)	1 (1.05)	0.261	1
Anterior and/or Lateral wall (%)	113 (57.08)	45 (41.67)	53 (55.79)	4.039	0.133
Inferior and/or Posterior wall	105 (45.06)	60 (55.56)	40 (42.11)	4.437	0.109
Maximum ST segment elevation (mm)	3.16 ± 1.27	2.95 ± 1.08	3.11 ± 1.33	1.072	0.345
Q wave	96 (41.20)	38 (35.19)	41 (42.11)	1.573	0.455
Symptom to door time (min)					
Median (25–75th)	189.46 (130.51–392.26)	170.13 (110.50–337.12)	180.11 (104.39–387.25)	0.978	0.613
Door to balloon time (min)					
Median (25–75th)	123.42 (91.35–210.92)	135.56 (80.49–198.55)	142.6 (98.53–228.11)	3.071	0.215
Door to needle time (min)					
Median (25–75th)	58.21 (28.20–110.83)	50.55 (31.02–126.02)	63.48 (32.01–130.44)	4.335	0.114
Left ventricular ejection fraction (%)	50.11 ± 13.60	48.92 ± 14.02	44.38 ± 13.05^ab^	6.051	0.003
IABP use	3 (1.29)	5 (4.63)	1 (1.05)	3.882	0.143
Successful PCI	166 (71.24)	73 (67.59)	57 (60.00)	3.92	0.141
Successful thrombolysis	40 (17.17)	21 (19.44)	18 (18.95)	0.314	0.855
Serum creatinine (mmol/l)	78.12 ± 31.13	85.47 ± 44.90	116.67 ± 59.01^ab^	28.612	<0.001
Peak troponin (ng/ml)	31.06 ± 16.28	30.19 ± 18.73	45.33 ± 15.26^ab^	28.253	<0.001
Hospitalization days	8.53 ± 4.78	8.36 ± 5.11	12.05 ± 5.74^ab^	18.401	<0.001

*BMI* body mass index, *HR* heart rate, *MI* myocardial infarction, *PCI* percutaneous coronary intervention, *IABP* intra-aortic balloon pump

^a^compared to Group A, *p* < 0.05

^b^compared to Group B, *p* < 0.05

### Clinical outcomes

The mortality rates of the patients in the Normal group, Moderate group and RI group were 1.72 %, 3.7 %, and 7.37 %, respectively, during hospitalization. Patients in the RI group had a significantly higher mortality rate compared with Normal group during hospitalization (*p* = 0.045). There were no significant differences in RMI, TVR, stroke and bleeding between the three groups. In-hospital MACE developed more frequently in the patients in the RI group compared with Normal group (*p* = 0.022). But there was no significant difference in the incidence of CIN (Table 2).

**Table 2:** Outcomes of patients according to eGFR group

	Normal group	Moderate group	RI group	*χ* ^2^	*p* value
	(*n* = 233)	(*n* = 108)	(*n* = 95)		
In hospital					
MACE	7 (3.00)	6 (5.56)	10 (10.53)^a^	7.664	0.022
death	4 (1.72)	4 (3.70)	7 (7.37)^a^	6.127	0.045
Recurrent MI	2 (0.86)	1 (0.93)	1 (1.05)	0.472	1
TVR	0	1 (0.93)	1 (1.05)	2.941	0.216
Stroke	0	0	0		NS
Bleeding	1 (0.43)	0	1 (1.05)	1.403	0.45
CIN	0	1 (0.93)	2 (2.11)	4.383	0.06
At 1-year follow-up					
MACE	35 (15.02)	24 (22.22)	36 (37.89)^ab^	20.734	<0.001
death	10 (4.29)	8 (7.41)	16 (16.84)^a^	14.814	<0.001
Recurrent MI	9 (3.86)	4 (3.70)	3 (3.16)	0.117	1
TVR	12 (5.15)	9 (8.33)	12 (12.63)	5.519	0.063
Stroke	1 (0.43)	2 (1.85)	1 (1.05)	2.02	0.424
Bleeding	3 (1.29)	1 (0.93)	4 (4.21)	3.244	0.173
At 2-year follow-up					
MACE	52 (22.32)	39 (36.11)^a^	50 (52.63)^a^	29.275	<0.001
death	16 (6.87)	11 (10.19)	23 (24.21)^ab^	20.227	<0.001
Recurrent MI	13 (5.58)	10 (9.26)	6 (6.32)	1.631	0.442
TVR	17 (7.30)	12 (11.11)	13 (13.68)	3.524	0.172
Stroke	3 (1.29)	3 (2.78)	3 (3.16)	1.919	0.391
Bleeding	3 (1.29)	3 (2.78)	5 (5.26)	4.275	0.109

*eGFR* estimated glomerular filtration rate, *MI* myocardial infarction, *TVR* target vessel revascularization, *MACE* major adverse cardiac events

^a^compared to Normal group, *p* < 0.05

^**b**^compared to Moderate group, *p* < 0.05

A similar trend was observed during 1 year of follow up after hospital discharge. Patients in the RI group had a significantly higher mortality rate compared with those in the Normal group (16.84 % v.s.4.29 %, *p* < 0.001), and a significantly higher MACE rate compared with the other two groups (*p* < 0.001) (Table 2).

During 2 years of follow up, patients in the RI group had a significantly higher mortality rate compared with the other two groups (24.21 % v.s. 6.87 % and 10.19 %, *p* < 0.001). There were no significant differences in recurrent myocardial infarction (RMI), target vessel revascularization (TVR), stroke and bleeding between the three groups. Compared with the RI group respectively, the Moderate group and the Normal group had higher MACE rates (36.11 % v.s. 22.32 %, 52.63 % v.s. 22.32 %, *p* < 0.001) (Table 2).

During 2 years of follow-up, there were 1 case of moderate bleeding and 4 cases of minor bleeding in the RI group. There were 3 cases of minor bleeding in the other two groups, respectively.

### Risk factors of 2 year mortality

**Fig. 2:**
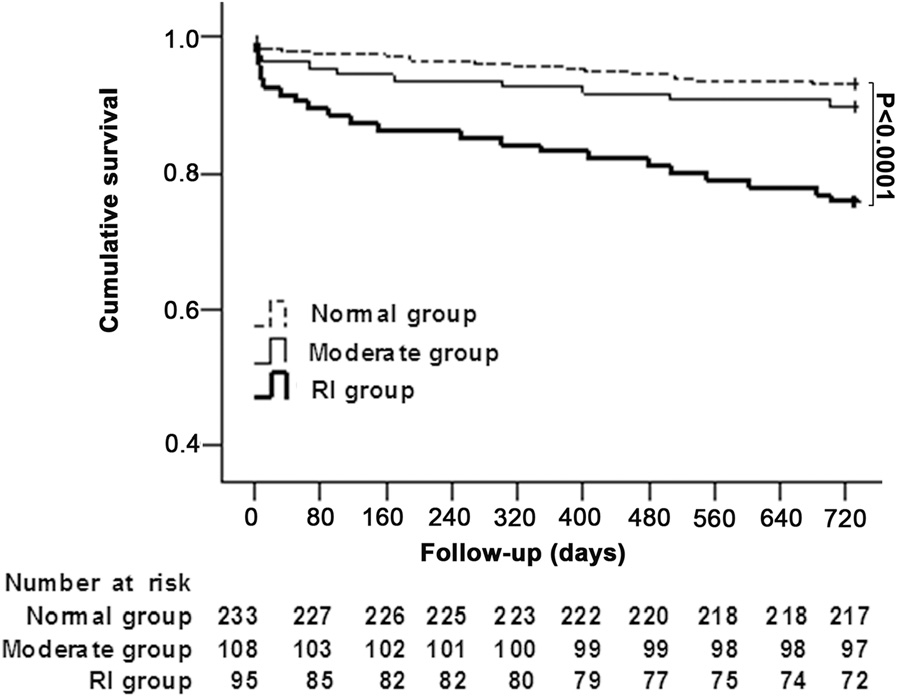
Kaplan-Meier curve survival analysis of the three groups of patients to 2-year post treatment. A represents the Normal group, B represents the Moderate group and C represents the RI group

Variables were analyzed by univariate analysis for significant factors for 2 year mortality and are presented in Table 3, this suggested that eGFR of less than 90 ml/min/1.73 m^2^, may be a predictor of 2 year mortality as both 60–90 ml/mim/ m^2^ eGFR and eGFR <60 ml/min/1.73 m^2^ were significant (both *p* > 0.001). Age ≥75 years was another significant factor (*p* = 0.049) as well as hypertension (*p* = 0.013), Killip class ≥2 (*p* = 0.023), and EF <40 (*p* = 0.025). The Kaplan–Meier survival curves are depicted in Fig. 2. The survival rate of the RI group was significantly lower than in the other two groups (log-rank test, *p* < 0.001).

**Table 3:** Univariate and multivariate analysis for prediction of 2-year mortality

	Univariate			Multivariate		
	HR	95%CI	*p* value	HR	95%CI	*p* value
eGFR ml/min/1.73 m^2^						
≥ 90	1			1		
60–90	2.911	1.295–3.731	<0.001*	2.081	1.250–2.842	<0.001*
< 60	5.043	1.585–8.960	<0.001*	3.872	2.004–6.131	<0.001*
Age ≥75	1.368	1.023–1.909	0.049*	1.461	1.011–1.952	0.024*
Diabetes	1.332	0.727–1.936	0.251			
Hypertension	1.241	1.032–1.453	0.013*	1.191	0.904–1.395	0.114
Hyperlipidemia	0.927	0.544–1.147	0.690			
Killip ≥ 2	1.593	1.032–2.301	0.023*	1.131	0.781–1.893	0.586
EF < 40 %	1.227	1.012–1.447	0.025*	0.905	0.451–1.060	0.647

*CI* confidence interval, *eGFR* estimated glomerular filtration rate, *EF* ejection fraction, *HR* hazard ratio

*p <0.05

Multivariate analysis then identified eGFR <90 ml/min/1.73 m^2^ and age ≥75 years as independent predictors of mortality at 2 years. In detail these were eGFR 60–90 ml/min/1.73 m^2^ with HR 2.081, 95%CI 1.250–2.842, *p* < 0.001; eGFR <60 ml/min/1.73 m^2^ with HR 3.872, 95%CI 2.004–6.131, *p* < 0.001; age ≥75 with HR 1.461, 95%CI 1.011–1.952, *p* = 0.024.

### Risk factors for in hospital mortality

All variables were also included into univariate analysis to investigate the independent predictors for in hospital mortality. The independent predictors of mortality in hospital were eGFR <60 ml/min/1.73 m^2^ and Killip ≥ 2 (Table 4). Further analysis of the significant factors by multiple cox proportional hazard modeling identified independent predictors of mortality. This showed that the independent predictors of hospital mortality were eGFR <60 ml/min/1.73 m^2^ [HR 1.832, 95 % CI 1.017–3.091, *p* = 0.033] and Killip ≥ 2 [HR 1.340, 95 % CI 1.012–1.647, *p* = 0.018].

**Table 4:** Univariate and multivariate analysis for prediction of in hospital mortality

	Univariate			Multivariate		
	HR	95%CI	*p* value	HR	95%CI	*p* value
eGFR ml/min/1.73 m^2^						
≥ 90	1			1		
60–90	0.942	0.653–1.131	0.670	0.865	0.586–1.390	0.220
< 60	2.655	1.147–4.238	0.003*	1.832	1.017–3.091	0.033*
Age ≥75	0.815	0.462–1.536	0.504			
Diabetes	0.893	0.601–1.072	0.443			
Hypertension	0.747	0.352–1.167	0.340			
Hyperlipidemia	0.86	0.317–1.053	0.622			
Killip ≥ 2	1.446	1.009–2.090	0.047*	1.34	1.012–1.647	0.018*
EF < 40 %	0.871	0.592–1.017	0.317			

*CI* confidence interval, *eGFR* estimated glomerular filtration rate, *EF* ejection fraction, *HR* hazard ratio

*p < 0.05

## Discussion

The aim of this study was to investigate the effect of RI on the mortality of female rural patients with STEMI. We demonstrated that the in hospitalization and 2-year mortality in the RI group (<60 ml/min/1.73 m^2^) were significantly higher than in the other two groups. And RI (eGFR <60 ml/min/1.73 m^2^) was an independent predictor of in hospital and 2-year mortality in the female STEMI patients after emergency reperfusion therapy. Compared with the Normal group, there was a 1.8-fold higher risk of death during hospitalization, and 3.9-fold higher risk of 2-year mortality. In the Moderate group (60–89 ml/min/1.73 m^2^), there was a 2.1-fold higher risk of 2-year mortality. We found a decreased eGFR was associated with a higher risk of death. Thus, RI was associated with higher rates of death. The most important finding of our study is that the inclusion of RI in the risk model improves the risk in female patients with STEMI in rural areas. We demonstrated hospitalization mortality in the RI group was 7.37 %. Another reported study showed a similar mortality rate of 7.69 % [25]. It has also previously been confirmed that RI is an independent risk factor for poor prognosis in both the short and long term for female STEMI patients [26].

Renal dysfunction is an independent risk factor for death in patients with STEMI [27]. And patients with renal dysfunction have been shown to have a 6 to 11-fold higher in-hospital mortality rate compared to patients with normal renal function [8]. These patients more commonly developed low left ventricular ejection fraction, higher Killip class, cardiogenic shock, hemodynamic instability or malignant arrhythmia during admission [28]. Previous studies have also found that the risk of MACEs and cardiac death at both 1 month and 1 year increased with lower eGFR [5]. Thus, the results of our study are in agreement with the previous research.

Comparison between the three groups showed that there were higher proportions of Killip class ≥ 2, longer hospital stay, low LVEF values, high serum creatinine levels and cTnI peak in the RI group. The patients had larger myocardial infarction area and worse heart function. These differences may be related to the high mortality in the RI group. The Killip class is a useful prognostic tool for predicting in hospital mortality [29], and our results support this as the ≥ class 2 was also a predictive factor for in hospital mortality by multivariate analysis. Another recent study on RI in STEMI also found a higher Killip class was found with RI, and those patients also showed increased mortality with RI [30]. That study identified that RI patients were more likely to be female as well as older, and more likely to have diabetes mellitus, and hypertension [30]. Our study also identified age, diabetes and hypertension as likely to be higher in the RI group. Increased Killip score and lower LVEF were also significant in RI patients in a study evaluating the in-hospital outcome of patients with acute STEMI [31]. The mean LVEF was also decreased in the RI patients in our study. In terms of the 2-year mortality risk factors the RI group was found to be at much higher risk than the other two groups in this study and the only other predictive factor was age ≥75 years. That was possibly an expected result [32].

We found there were no long-term standardized medication regimens after hospital discharge in the RI group. And dual antiplatelet therapy was used less in hospital in this group. Underuse of antiplatelet therapy, ACE inhibitors, β-blockers and statins might also be related to a reduced survival rate in patients with renal insufficiency as discontinuation of cardiac medication is itself associated with increased risk of mortality [33]. This may be another factor that is related to the high mortality in the RI group.

Renal dysfunction was associated with a 3 fold increased odds of discontinuation of antiplatelet drugs in patients with PCI [34]. Reasons for shorter duration of antiplatelet therapy and discontinuation observed in these studies and ours may include bleeding events, scheduled invasive procedures, psychiatric drug use, unemployment, patient choice and non-adherence and other medical events not specified including earlier mortality. It had been reported AMI patients with decreased GFR may receive less aggressive evidence-based therapies as those normal patients [35].

This study has some limitations as it was a prospective multicenter study with a limited sample size because of the limited number of rural patients. In the RI group the patients were not grouped further to eGFR 30–60 ml/min/1.73 m^2^ and eGFR <30 ml/min/1.73 m^2^ to provide information on the severity of RI. We also were unable to provide a mean value of eGFR for the groups in this study because some of the raw data was lost, so we had to rely on the grouping ranges for our analysis. We did not include a control group without cardiac disease, to investigate whether these results relating to RI and mortality would be similar in a group of patients without STEMI. In addition, we did not address the question of a relationship between cardiac disease and kidney disease in these patients. Further analysis of more pathophysiological factors such as biomarkers for cardiac damage for example troponin would provide important information on the relationship between cardiac and kidney diseases.

## Conclusions

In this real-world prospective multicenter study we found among female rural patients with STEMI after thrombolytic therapy or primary PCI therapy that RI was an independent risk factor for in-hospital and long-term mortality and was associated with poor prognosis. RI could be one of the better indices for clinical risk stratification.

## Availability of supporting data

The data set supporting the results of this article are included within the article.

## Ethics approval and consent to participate

This study was conducted in accordance with the declaration of Helsinki, and was conducted with approval from the Ethics Committee of China Medical University. Written informed consent was obtained from all participants.

## Consent for publication

Not applicable.

## Appendix 1

### Details of medication

There were no significant difference in the administration of aspirin, ACEI/ARB, statins, beta -blockers, LWMH and traditional Chinese medicine between the three groups. But clopidogrel and dual antiplatelet therapy were lower in the RI group compared with the Normal group during hospitalization. The patients were followed up at 2 year. We found that clopidogrel, dual antiplatelet therapy, ACEI/ARB, statins, b-blocker were less used,but traditional Chinese medicine were more used in the RI group during follow-up (Appendix 1).

**Table 5:** Medication use

	Normal group (*n* = 233)	Moderate group (*n* = 108)	RI group (*n* = 95)
In hospital			
Aspirin	233	107	95
Clopidogrel	233	108	92
dual antiplatelet therapy	233	107	92
ACEI/ARB	221	101	83
Statins	205	89	77
Beta blockers	209	98	79
Low molecular heparin	230	107	91
Traditional Chinese medicine	157	61	59
At 2-year follow-up			
Aspirin	176	84	55
Clopidogrel	61	29	8
dual antiplatelet therapy	60	29	8
ACEI/ARB	63	3	7
Statins	25	14	9
Beta blockers	155	58	44
Traditional Chinese medicine	81	41	50

## Abbreviations

ACE: angiotensin-converting enzyme
ARB: angiotensin receptor antagonist
BMI: body mass index
CI: confidence intervals
CIN: contrast-induced nephropathy
CKD-EPI: chronic kidney disease epidemiology collaboration
CKMB: creatine kinase MB
Ctni: cardiac troponin I
ECG: electrocardiogram
EF: ejection fraction
eGFR: estimated glomerular filtration rate
GUSTO: global use of strategies to open occluded coronary arteries
HDL: high-density lipoprotein
HR: hazard ratio
IQR: interquartile range
KDIGO: kidney disease: improving global outcomes
LDL: low-density lipoprotein
MACE: major adverse cardiac events
MDRD: modification of diet in renal disease
PCI: percutaneous coronary intervention
RI: renal insufficiency
RMI: recurrent myocardial infarction
Scr: serum creatinine
SD: standard deviation
STEMI: ST-segment elevation acute myocardial infarction
TIMI: thrombolysis in myocardial infarction
TVR: target vessel revascularization
WBC: white blood cell
